# Intensity and Duration of Negative Emotions: Comparing the Role of Appraisals and Regulation Strategies

**DOI:** 10.1371/journal.pone.0092410

**Published:** 2014-03-26

**Authors:** Karen Brans, Philippe Verduyn

**Affiliations:** Faculty of Psychological and Education Sciences, KU Leuven, Leuven, Brussels, Belgium; Brock University, Canada

## Abstract

Intensity and duration are two central characteristics of an emotional response. Appraisals and regulation strategies are among the most important determinants of these emotion features. However, as intensity and duration are only moderately correlated, appraisals and regulation strategies may be differently related to these characteristics. A systematic empirical study comparing predictors of emotion intensity and duration is missing. The goal of the present study is to fill this gap. Participants were asked to recall recently experienced episodes of anger, fear, disgust, guilt, sadness, and shame. Subsequently, they were asked to answer a number of questions regarding (a) the intensity and duration of these emotions, (b) their appraisal of the emotion-eliciting event, and (c) their use of a wide range of regulation strategies. Emotion intensity was found to be mainly predicted by appraisals whereas emotion duration was equally well predicted by appraisals and regulation strategies.

## Introduction

Each emotional episode is characterized by two stages [1]–[2]. During the first stage the emotion blossoms and strengthens over time, adding to the overall intensity of the emotion. During the second stage the emotion fades with the speed of this recovery process being strongly related to the duration of the emotion.

Intensity and duration are two salient features of an emotional response [3]. Indeed, when people talk about their emotions, they often not only describe the nature of the emotion but also the intensity or duration (e.g., I was *very* angry, I felt sad *all day long*). The study of emotion intensity and duration is not only important to get a better understanding of the emotional life of normal individuals but it is also of high importance in clinical settings as emotional disturbances are often characterized by inappropriately strong (or weak) and long (or short) emotions [4].

Emotions display a remarkable variability in intensity and duration [5]–[8]. Consequently, one may wonder which determinants account for this variability. In the next two sections, we will discuss research on the two major classes of predictors of emotion intensity and duration, namely appraisals and emotion-regulation strategies.

### Predictors of Emotion Intensity

Frijda and colleagues [9] argued that appraisals and emotion-regulation strategies are the most important predictors of emotion intensity. Appraisals refer to evaluations of the emotion-eliciting event. Several different appraisal dimensions have been proposed (for a comparative overview, see [10]), many of them entailing a comparison between an event and a desired state. For example, an appraisal of goal congruency implies a check of whether an event is conducive to reaching one’s goals. A central hypothesis within appraisal theory is that, when an event is appraised to create a mismatch between the current state and a desired one, a negative emotion follows. Moreover, the stronger this mismatch, the more intense the ensuing negative emotion will be [11]–[12]. This hypothesis has been supported by a number of studies [9], [13].

Emotion regulation has been defined as the strategies people use to influence which emotions they have, when they have them, and how they experience and express them [14]. The relation between emotion and regulation strategies is complex as, on the one hand, emotion intensity determines the required amount of regulation and, on the other hand, regulation will influence emotion intensity [9]. Regarding the former, high emotional intensity has been found to initiate an increased use of regulation strategies regardless of the nature of the strategy [15]. Regarding the latter, it has been shown that some strategies (e.g., rumination) increase emotion intensity whereas others (e.g., reappraisal) dampen it [14], [16].

In most studies on determinants of emotion intensity, appraisals and regulation strategies were investigated separately. Consequently, it remains largely unclear which class of predictors explains most variability in emotion intensity. A notable exception is a study by Sonnemans & Frijda [9] in which it was found that appraisals were more predictive of emotion intensity than regulation strategies. This relatively low predictive power of regulation strategies may, however, be partly due to the way emotion regulation was assessed. Specifically, for each recalled emotion episode, participants were asked to indicate to what extent they had tried to dampen their emotional feelings, behavior, and expression. As such, regulatory effort was measured but the nature of the particular regulation strategy that was deployed was not taken into account. This is troublesome as the relationship between emotion regulation and emotion intensity depends on the nature of the regulation strategy in question [14]. Consequently it remains to be seen whether appraisals still explain more variance in intensity than regulation when the latter is measured in a more differentiated fashion.

### Determinants of Emotion Duration

For a long time research on emotion duration was rather scarce. However, during the last two decades several attempts have been made to contribute solid evidence regarding predictors of emotion duration. In a recent paper, Van Mechelen, Verduyn, & Brans [17] reviewed determinants of duration and concluded that, similarly to intensity, appraisals and emotion-regulation strategies are among the most important predictors of emotion duration.

The relation between appraisals and emotion duration has been empirically demonstrated in a number of recent studies. In particular, Verduyn et al. [6] found that perceived event importance is positively related to emotion duration. Furthermore, it has been shown that negative emotions last especially long when the eliciting event and its consequences are perceived to be incongruent with the individual’s goals, values, and self-ideal, creating a mismatch [18].

The relation between several types of emotion-regulation strategies and emotion duration has also been studied ([6], [19]). In these studies rumination has been found to sustain emotions [6], [16] whereas reappraisal and distraction shorten emotion duration [19].

However, similarly to emotion intensity, these determinant classes were typically not studied simultaneously. Consequently, the relative predictive power of the different classes of predictors is unclear. Importantly, as intensity and duration have been found to correlate only moderately [5], [18], [20] it cannot be taken for granted that the predictive power of the different classes of determinants is similar for duration and intensity. A systematic empirical study is needed to directly compare the degree to which appraisals and regulation strategies are predictive of emotion intensity and of duration.

### The Present Study

The overall aim of the present study is to systematically investigate appraisals and regulation strategies as predictors of the intensity and the duration of negative emotions. This will contribute to a better understanding of the factors underlying variability in emotion intensity and duration. Regarding appraisals, dimensions were selected from the Geneva Appraisal Questionnaire [21]: Importance and disadvantageousness of the emotion-eliciting event, other and own responsibility, problem- and emotion-focused coping, expectedness, impact of the event on the self-image, and, injustice and immorality of the event. With regard to emotion-regulation strategies, five commonly used strategies were investigated: Reappraisal, rumination, reflection, distraction, and expressive suppression. Regarding emotions, six commonly experienced negative emotions were selected [22]: Anger, disgust, fear, guilt, sadness, and shame.

Within our overall aim, we distinguish three subgoals with associated hypotheses: First, we aimed to verify whether appraisals and emotion-regulation strategies are indeed important predictors of emotion intensity and duration. In this regard, we hypothesized that each class of predictors would account for a substantial part of variability in emotion intensity and duration.

**Figure 1:**
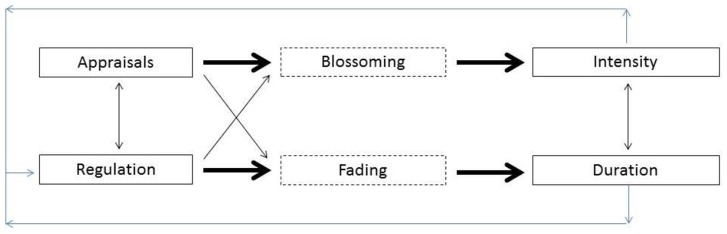
Graphical representation of hypothesized relationships between appraisals and regulation strategies, and the intensity and duration of emotions. Thick and thin lines represent strong and weak connections, respectively.

Second, because of the rather small correlation between intensity and duration, we aimed to investigate whether determinant classes are differentially related to emotion intensity and duration. Our hypotheses (for a graphical representation, see Figure 1) result from the timing of the two central stages of an emotional episode: emotion blossoming (stage 1 associated with emotion intensity) and emotion fading (stage 2 associated with emotion duration). As appraisal processes already start taking place from the onset of the emotional episode, we expect that they will be especially predictive of the blossoming process and associated emotion intensity. This hypothesis is consistent with the finding of Sonnemans & Frijda [5] that appraisals are highly predictive of emotion intensity. In contrast, emotion regulation comes especially into play later on in the episode, determining the steepness with which the emotional response returns to a neutral baseline [2], [23]. Consequently, regulation can be expected to be especially related to features reflecting emotional recovery such as emotion duration.

Even though appraisals are expected to be the main predictor of emotion intensity, we expect them to be related to emotion duration to some degree as well as certain appraisal configurations may slow down the recovery process. For example, event importance has been found to be predictive of emotion duration even when controlling for emotion intensity [6], [18] and low coping potential has been found to be associated with a prolongation of emotional recovery [24]. Similarly, we expect regulation strategies to be mainly predictive of emotion duration but also to some degree of emotion intensity. Emotion blossoming is not necessarily restricted to the period immediately following the emotion-eliciting event but may take some time [7] during which regulation strategies may stimulate (or hamper) the blossoming process, which in turn affects emotion intensity. For example, rumination has been found to intensify the emotional response following an emotion induction [25].

Third, we aimed to investigate how specific appraisals and regulation strategies are related to emotion intensity and duration. Regarding appraisals, we hypothesized that the more an event would be perceived as important and as creating a mismatch, the more intense and longer lasting the negative emotion would be [11], [12], [18]. Specifically, we expected importance and disadvantageousness of the event, impact of the event on the self-image, injustice and immorality of the event to be positively related to negative emotion intensity and duration, whereas emotion- and problem focused coping would be negatively related. Furthermore, as own and other responsibility do no not directly express a mismatch, they were hypothesized not to be related to negative emotion intensity and duration. Finally, in line with previous findings [9], [18] we hypothesized expectedness not to be related to negative emotion intensity and duration. With regard to regulation, we hypothesized that rumination and expressive suppression would be positively related to negative emotion intensity and duration [26], [27] whereas reflection, reappraisal and distraction would be negatively related to it ([14], [28], [29].

To test these hypotheses, participants were asked to recall recently experienced episodes of anger, fear, disgust, guilt, sadness, and shame. Subsequently, they were asked to answer a number of questions regarding (a) the intensity and duration of these emotions; (b) their appraisal of the emotion-eliciting event; and (c) their use of a wide range of emotion-regulation strategies.

## Methods

### Ethics Statement

The study was conducted in April 2011 as part of a large collective research program organized and approved by the Faculty of Psychology and Educational Sciences, KU Leuven, Belgium. All first year psychology students are invited to participate in this research in exchange for course credits. Written informed consent was obtained from all participants at the start of the program.

In accordance with the “Law of 7 May 2004 concerning experiments on the human person” an authorization is necessary from the Medical Ethics Committee of the University Hospitals Leuven, Faculty of Medicine for experiments that “touch the person in their essence.” These experiments mean studies which include physical changes such as the need to breathe faster, painful stimuli, using deception and other (taken from https://ppw.kuleuven.be/intern/ethischecommissie/index and translated to English). In the present study, participants completed a retrospective questionnaire and consequently the study falls outsides these conditions and did not necessitate approval from the ethics committee.

However, as of June 2012, the Faculty of Psychology and Educational Sciences has decided that all studies within the collective research program would require ethical approval. Since then, we have applied for and have been granted ethical approval for studies using similar participants and similar questionnaires.

Only one of the participants (N = 408) was a legal minor (The participant was 17 at the time of the data collection). As mentioned above, informed consent was obtained from all participants at the start of the collective research program (September 2010). There was no different procedure for this participant because according to the “Law of 22 August 2002 on Patient Rights” (Art.12§2) “The rights set out in this act may be exercised independently by the minor patient who is capable to reasonably assess/judge his interests.” (Taken from http://www.ejustice.just.fgov.be/cgi_loi/change_lg.pl?language=nl&la=N&cn=2002082245&table_name=wet and translated to English). It was assumed that minors aged 17 are capable to reasonably assess their interests.

### Participants

Participants were 408 first-year students of the University of Leuven with a mean age of 18.9 (*SD* = 1.32). The sample consisted of 73 men and 332 women (three participants did not report their gender). Participation was in partial fulfillment of a course requirement.

### Materials

For the present study, an emotion questionnaire was designed. This questionnaire was designed in Dutch. On the first page of this questionnaire, participants were explained that they were expected to recall a number of recent emotion episodes (anger, fear, disgust, guilt, sadness, and shame) and answer a number of questions on them. Subsequently, two important conceptual clarifications were offered. First, to make sure that participants would supply information regarding emotions and not moods, they were told that an emotion is always elicited by a certain internal or external event, and thus has a clear onset point [30]. Second, to avoid different interpretations of the concept of emotion duration, participants were told that an emotion ends as soon as an emotion is no longer felt for the first time (with, if the emotion is re-experienced later on, this is to be considered a new episode). As an exception, interruptions in the emotion episode due to sleep were allowed, making it possible for an emotion episode to last longer than a day. This definition has been repeatedly used in previous research on emotion duration [4], [6], [19].

The remainder of the questionnaire consisted of six two-page sections with each section corresponding to a different emotion. The order of these sections was randomized across participants. Each section started with the instruction to recall a recent episode of the emotion in question and to briefly describe the emotion-eliciting event. Subsequently, participants were asked to indicate when the emotion-eliciting event occurred (1 = a day ago, 2 = weeks ago, 3 = months ago, or 4 = years ago). Next, they were asked to rate the global intensity and the duration of the emotion episode. For intensity, they rated the overall intensity of the emotion on an 8-point Likert scale, ranging from 0 (*not intense at all*) to 7 (*very intense*). For duration, participants were asked to rate the duration of the emotion by specifying the number of days, hours, minutes and/or seconds the emotional experience had lasted.

Subsequently, the emotion-eliciting event was rated on a number of appraisal dimensions. For all the dimensions, the item started with ‘To which extent’ and was completed with, ‘was the event that elicited the emotion important to you?’ (importance); ‘was the event that elicited the emotion disadvantageous to you?’ (disadvantageousness); ‘did you think someone else was responsible for the occurrence of the event that elicited the emotion? (other responsibility)’; ‘did you ought yourself responsible for the occurrence of the event that elicited the emotion?’ (own responsibility); ‘did you think that you could change something about the event that elicited the emotion?’ (problem-focused coping); ‘did you think that you could deal with the emotions that were elicited by the event?’ (emotion-focused coping); ‘did you expect the event that elicited the emotion?’ (expectedness); ‘did the event have a negative impact on your self-image?’ (impact on self-image); ‘did you find the event that elicited the emotion unjust?’ (injustice); ‘did you think that the event that elicited the emotion was immoral?’ (immorality). All appraisal items were rated on an 8-point Likert scale ranging from 0 to 7, with 7 indicating strong agreement with the item.

Finally, participants were asked to indicate which emotion-regulation strategies they had deployed during the episode. For all five strategies under study, the item started with ‘At the time, when you experienced the emotion, to which extent did you’ and was completed with ‘ruminate about the emotion-eliciting event?’ (rumination), ‘calmly reflect on the emotion-eliciting event?’ (reflection), ‘try to see the emotion-eliciting event from a different perspective?’ (reappraisal), ‘try to suppress the expression of your emotion?’ (expressive suppression), and ‘divert your attention away from what happened?’ (distraction). Each strategy was rated on an 8-point Likert scale ranging from 0 (*not at all*) to 7 (*very much*).

### Procedure

Participants were invited to the psychology department and were asked to complete several different questionnaires. In the present study we report on the emotion questionnaire that was designed to investigate predictors of emotion intensity and duration described above. At the end of the study, participants were thanked for their participation.

## Results

### Descriptive Statistics

#### Time since occurrence

In line with the instructions, the majority of the reported episodes happened recently. In particular, on average, 73% percent of the episodes were experienced the previous day or within the last couple of weeks.

#### Intensity and duration

**Figure 2:**
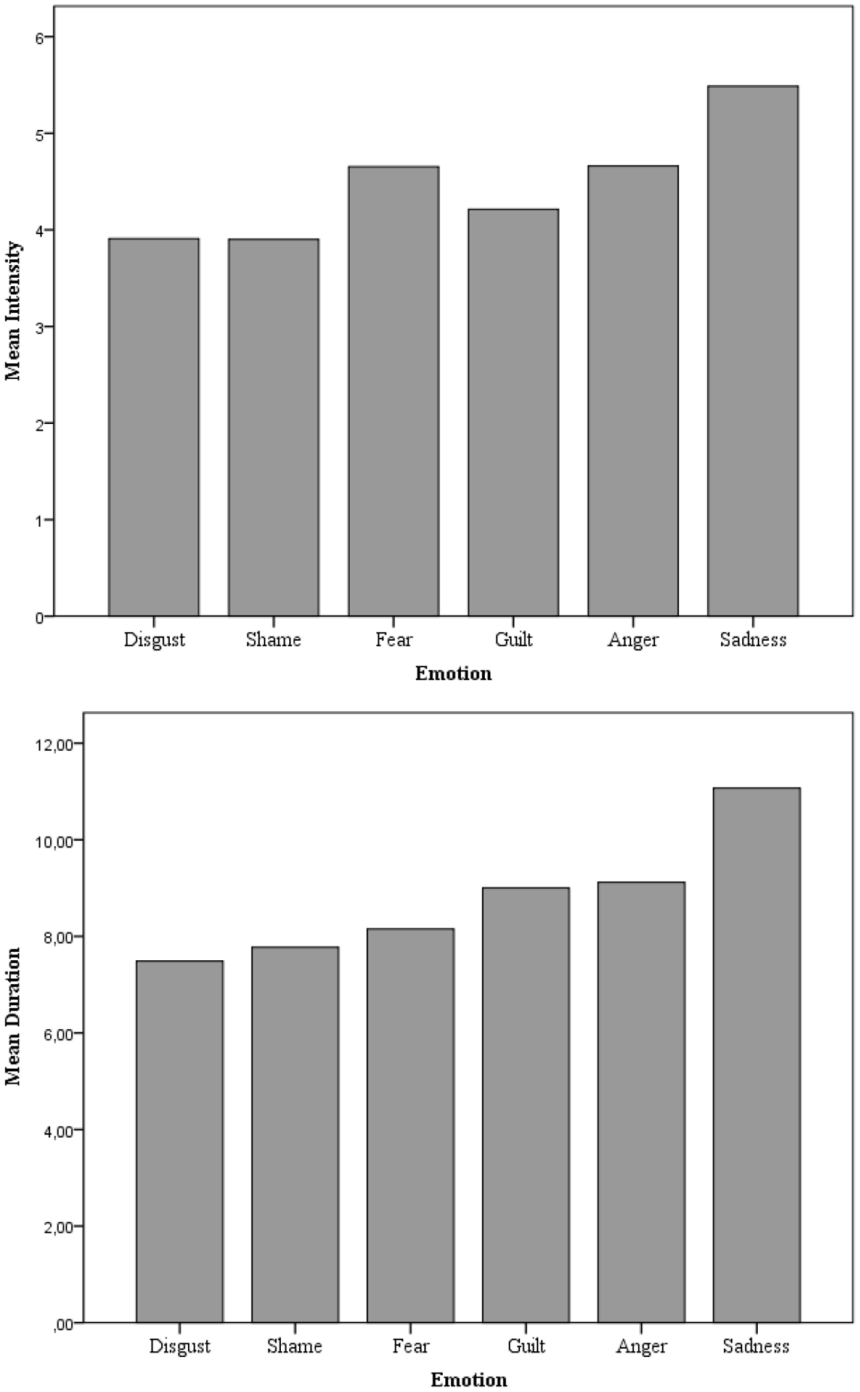
The mean intensity (top) and mean (log transformed) duration (bottom) by emotion.

Descriptive statistics for the intensity and duration are presented in Table 1 (for a graphical representation see Figure 2). Differences between emotions in intensity and duration were investigated by means of a Repeated Measures ANOVA. Subsequently, we examined all pair-wise comparisons (with Bonferroni correction) to get a more refined understanding of the nature of the between-emotion differences. Specific differences between emotion pairs can be read from Table 1. Below we will only mention the emotions with the highest and the lowest ratings.

**Table 1:** Descriptive Statistics (Means and Standard Deviations) for Dependent Variables: Intensity and (log transformed) Duration and the Correlations between Intensity and (log transformed) Duration.

	Sadness	Anger	Guilt	Fear	Shame	Disgust
Intensity						
Mean	5.49_a_	4.66_b_	4.21_c_	4.65_b_	3.90_c_	3.91_c_
* SD*	1.44	1.59	1.75	1.77	1.76	1.95
Duration						
Mean	11.07_a_	9.12_b_	9.00_b_	8.15_c_	7.78_c_	7.49_c_
* SD*	2.38	2.32	2.40	2.77	2.65	2.61
Correlation	0.38	0.32	0.41	0.27	0.40	0.38

*Note.* Within rows, emotions sharing a subscript do not differ significantly. All correlations were significant at *p*<.001.

Evidence for variability in intensity between emotions was found (*F*(4.86,1677.65) = 59.90; *p*<.001; partial *η^2^* = .15 ). It appeared that, on average, episodes of sadness were rated as most intense, and episodes of shame, guilt, and disgust were rated lowest in intensity.

Regarding duration, a highly positively skewed distribution was obtained. Therefore, to avoid that outliers (i.e., very long emotional episodes) would bias our findings, the duration ratings (in seconds) were logarithmically transformed. Evidence for variability in duration between emotions was found (*F*(4.93,1569.00) = 104.79; *p*<.001; partial *η^2^* = .25). It appeared that, overall, episodes of sadness lasted the longest, and episodes of fear, shame, and disgust were the shortest.

Intensity and (the log transformed) duration variable showed small to moderate correlations. In particular, the correlation ranged from .27 (fear), up to .41 (guilt), with a median correlation of.38.

#### Determinants

In Table 2, for each emotion, the means and standard deviations for the different appraisal dimensions and regulation strategies are presented. To investigate possible differences between emotions, the same data analytic strategy as for intensity and duration was used.

**Table 2:** Means and Standard Deviations for the Different Classes of Determinants.

	Anger		Fear		Sadness		Shame		Guilt		Disgust	
	Mean	*SD*	Mean	*SD*	Mean	*SD*	Mean	*SD*	Mean	*SD*	Mean	*SD*
*Appraisals*												
Importance	4.66_b_	2.06	3.78_c_	2.45	5.80_a_	1.59	3.39_c_	2.26	4.49_b_	2.02	2.49_d_	2.38
Disadvantageousness	4.26_a.b_	2.17	3.77_c_	2.35	4.55_a_	2.27	4.00_b,c_	2.12	3.63_c_	2.28	2.35_d_	2.45
Other responsibility	5.82_a_	1.82	3.08_c_	2.81	3.55_b_	2.71	1.87_d_	2.39	1.73_d_	2.21	5.75_a_	1.98
Own responsibility	2.02_d_	2.10	2.91_c_	2.57	2.53_c_	2.48	5.34_b_	2.02	5.79_a_	1.58	0.99_e_	1.72
Problem-foc. coping	2.45_c_	2.24	2.63_c_	2.42	2.22_c_	2.32	3.71_b_	2.35	4.68_a_	2.20	1.52_d_	1.95
Emotion-foc. coping	4.29_b_	1.86	4.07_b_	1.92	3.36_c_	1.92	4.77_a_	1.82	4.67_a_	1.71	4.90_a_	1.96
Expectedness	2.74_a_	2.19	2.91_a_	2.54	3.16_a_	2.42	2.66_a_	2.31	2.77_a_	2.20	2.65_a_	2.35
Injustice	4.97_a_	2.01	2.54_d,e_	2.51	4.27_b_	2.48	2.13_e_	2.14	2.68_d_	2.28	3.27_c_	2.67
Self-image	2.74_b_	2.40	2.10_c_	2.24	2.99_b_	2.52	3.65_a_	2.24	3.23_a,b_	2.27	1.25_d_	2.00
Immorality	3.11_a_	2.32	1.89_c_	2.22	2.57_b_	2.30	1.70_c_	1.98	2.28_b_	2.08	3.06_a_	2.57
*Regulation*												
Rumination	4.20_b_	2.39	3.77_b,c_	2.48	5.43_a_	1.77	3.79_b,c_	2.43	4.49_b_	2.15	2.31_d_	2.55
Reflection	3.35 _b,c_	2.25	3.02_c_	2.34	4.42_a_	2.18	3.00_c_	2.36	3.67_b_	2.23	2.20_d_	2.31
Reappraisal	2.65_a,b_	2.15	2.22_c_	2.23	3.00_a_	2.26	2.36_b,c_	2.28	2.67_a,b_	2.20	1.51_d_	1.98
Suppression	3.32_b,c_	2.33	3.58_a,b_	2.48	3.81_a_	2.24	3.44_a,b_	2.38	3.05_c_	2.27	2.37_d_	2.37
Distraction	3.48_c_	2.32	4.13_a_	2.45	4.34_a_	2.20	4.06_a,b_	2.36	3.61_b,c_	2.33	3.32_c_	2.47

*Note.* Within rows, emotions sharing a subscript do not differ significantly.

Firstly, from Table 2 it appears that, for all appraisal dimensions, except for expectedness, there were significant differences between emotions (for importance, *F*(4.76, 1638.31) = 116.87; partial *η^2^* = .25; for disadvantageousness, *F*(4.89,1693.53) = 50.23; partial *η^2^* = .13; for other responsibility *F*(4.70,1626.05) = 233.23; partial *η^2^* = .40; for own responsibility, *F*(4.65,1604.45) = 298.49; partial *η^2^* = .46; for problem-focused coping *F*(4.84,1668.12) = 102.19; partial *η^2^* = .23; for emotion-focused coping *F*(4.80,1657.52) = 42.91; partial *η^2^* = .11; for injustice, *F*(4.88,1688.65) = 96.02; partial *η^2^* = .22; for impact on the self-image, *F*(4.81,1653.99) = 63.72; partial *η^2^* = .16; for immorality (*F*(4.57,1554.85) = 30.76; partial *η^2^* = .08, all *p*s <.001). For expectedness, there were no differences between emotions (*F*(4.93,1705.07) = 1.99; *p*>.05).

Secondly, from Table 2 it appears that, for all strategies, there were significant differences between emotions (for rumination *F*(4.80,1656.66) = 84.71; partial *η^2^* = .20; for reflection *F*(4.99,1725.42) = 50.33; partial *η^2^* = .13; for reappraisal *F*(4.95,1713.95) = 28.17; partial *η^2^* = .08; for suppression *F*(4.92,1703.92) = 21.49; partial *η^2^* = .06; for distraction *F*(4.83, 1672.55) = 12.60; partial *η^2^* = .04; all *ps* <.001). Interestingly, it appeared that for all strategies regulatory effort was highest in case of sadness and lowest in case of disgust.

Finally, we also investigated the correlations between appraisals and regulation strategies for each emotion separately (see Tables S1–S6 in File S1). The results largely generalized across emotions, except for sadness where relationships were found to be somewhat less outspoken. It appeared that a) importance, advantageousness, impact on self-image, and immorality were significantly and positively associated with the use of emotion-regulation strategies, and b) emotion-focused coping was significantly and negatively associated with the use of emotion-regulation strategies. For the other appraisal dimensions, the results varied depending on the specific regulation strategy or emotion under study.

### Regression Analyses

#### Strength of associations: effect sizes

Table 3 reflects the amount of variability in intensity (or duration) explained by (a) all appraisals and regulation strategies simultaneously; (b) all appraisals simultaneously (with and without controlling for regulation strategies); and (c) all regulation strategies simultaneously (with and without controlling for all appraisal strategies).

**Table 3:** Proportion of Variability in Intensity and Duration Explained by Appraisals and Emotion-regulation Strategies simultaneously, by Appraisals with and without Controlling for Regulation, and by Regulation with and without Controlling for Appraisal.

		Emotion					
Predictors	Criterion	Anger	Fear	Sadness	Shame	Guilt	Disgust
*Appraisals and regulation*	Intensity	.33	.27	.32	.28	.43	.24
	Duration	.28	.29	.32	.33	.33	.36
*Appraisals*							
Not controlling for regulation	Intensity	.32	.26	.30	.25	.39	.21
Controlling for regulation	Intensity	.17	.15	.19	.07	.12	.07
Not controlling forregulation	Duration	.22	.23	.31	.28	.24	.33
Controlling for regulation	Duration	.04	.07	.23	.07	.06	.08
*Regulation*							
Not controlling forappraisals	Intensity	.15	.12	.12	.22	.30	.17
Controlling for appraisals	Intensity	.01	.01	.02	.03	.04	.02
Not controlling forappraisals	Duration	.24	.22	.09	.27	.27	.28
Controlling for appraisals	Duration	.06	.05	.01	.05	.09	.03

We first investigated the total percentage of variability explained. When intensity was predicted by all classes of determinants simultaneously, it appeared that the percentage of variability explained ranged from 24% (disgust) up to 43% (guilt), with a median of 30%. For duration, it appeared that, the percentage of variability explained by all classes of determinants jointly ranged from 28% (anger and fear) up to 36% (disgust) with a median of 33%.

Secondly, we investigated the percentage of variability explained by appraisals. For intensity, the percentage of variability explained by a model including all appraisal dimensions simultaneously, ranged from 21% (disgust) up to 39% (guilt) with a median of 28%. When investigating a model in which we first added all regulation strategies and in a second step all appraisal dimensions, it appeared that appraisals uniquely predicted between 7% (disgust and shame) and 19% (sadness) of variability in intensity with a median of 13%. For duration, the percentage of variability explained by the model including all appraisal dimensions simultaneously ranged from 22% (anger) up to 33% (disgust) with a median of 26%. When investigating a model in which we first added all regulation strategies and in a second step all appraisal dimensions, it appeared that appraisals uniquely predicted between 4% (anger) and 23% (sadness) of variability in duration with a median of 7%.

Finally, we investigated the percentage of variability explained by emotion-regulation strategies. For intensity, the percentage of variability explained by a model including all emotion-regulation strategies ranged from 12% (fear and sadness) up to 30% (guilt) with a median of 16%. When investigating a model in which we added all appraisal dimensions in a first step and all regulation strategies in a second step, it appeared that regulation uniquely predicted between 1% (fear and anger) and 4% (guilt) of variability in intensity with a median of 2%.

For duration, the percentage of variability explained by a model including all emotion-regulation strategies ranged from 9% (sadness) up to 28% (disgust) with a median of 25%. When investigating a model in which first all appraisals dimensions and then all regulation strategies it appeared that regulation uniquely predicted between 1% (sadness) and 9% (guilt) of variability in duration with a median of 5%.

#### Direction of association: regression weights

The standardized regression weights obtained when predicting emotional intensity or duration by all appraisal dimensions simultaneously are presented in Table 4. To ease readability only significant regression weights are displayed (the complete table can be found in Table S7 in File S2).

**Table 4:** Standardized Regression Weights for the Appraisals when Predicting the Intensity and Duration of Emotion Episodes.

	Sadness		Anger		Guilt		Fear		Shame		Disgust	
	Intensity	Duration	Intensity	Duration	Intensity	Duration	Intensity	Duration	Intensity	Duration	Intensity	Duration
Importance	0.35***	0.21***	0.36***	0.28***	0.37***	0.29***	0.31***	0.31***	0.22***	0.32***	0.39***	0.38***
Disadvantage	0.13*	0.26***	0.12*		0.10*		0.20***		0.15**			
Responsibility												
Other		−0.12*										
Own												
Coping												
Problem-foc.		−0.15**			0.12**			−0.10^†^			−0.09^†^	
Emotion- foc.	−0.17***	−0.15**	−0.25***	−0.12*	−0.14**	−0.19***	−0.17***			−0.15**		0.09^†^
Expectedness								0.11*	−0.08^†^			
Injustice	0.11*	0.12*										
Self-image				0.13*	0.16**	0.10†		0.12*	0.18**	0.12*		0.20**
Immorality		0.09^†^		0.09^†^	0.12*					0.11*		

*Note*. ***p<.001 **p<.01 *p<.05 ^†^ *p*<.1.

*Note*. Only significant regression weights are displayed in the table.

When predicting emotion intensity by all appraisal dimensions simultaneously, it was found that, for all emotions under study, importance of the emotion-eliciting event was positively related to emotion intensity. Moreover, in line with the mismatch hypothesis, for almost all emotions (except for disgust) it appeared that the more disadvantageous the emotion-eliciting event was perceived, the more intense the emotion was. Also, for almost all emotions under study (except for shame and disgust), emotion-focused coping was significantly negatively related to intensity, suggesting that the more one could cope with the emotions elicited by the event, the less intense the emotion was. For the other appraisal dimensions expressing a mismatch (immorality, self-image, injustice) some of the expected relations were found, but they did not generalize across emotions. For example, events with negative consequences for the self-image were positively associated with emotion intensity but only in case of guilt and shame. Finally, as expected it was found that expectedness, and, own and other responsibility did not predict intensity.

When predicting emotion duration by all appraisal dimensions simultaneously, it was found that, for all emotions under study, importance of the emotion-eliciting event was positively associated with emotion duration. Furthermore, in line with the mismatch hypothesis, it appeared that for almost all emotions under study (except for sadness) the more an event had a negative impact on one’s self-image the longer the emotion lasted. Moreover, for all emotions (except for fear) it appeared that emotion-focused coping was associated negatively with emotion duration. For the other appraisal dimensions expressing a mismatch (disadvantageousness, immorality, and injustice) some of the expected relations were found, but these did not generalize across emotions. For example, immorality was positively related to the duration of sadness, anger, and shame. Finally, similar to the findings for intensity, expectedness did not predict duration (except for fear). Also, other and own responsibility were not related to the duration of emotion episodes.

The standardized regression weights obtained when predicting emotion intensity or duration by all regulation strategies simultaneously are presented in Table 5. To ease readability only significant regression weights are displayed in the table (the complete table can be found in Table S8 in File S2).

**Table 5:** Standardized Regression Weights for the Emotion-regulation strategies when Predicting Intensity and Duration of Emotion Episodes.

	Sadness		Anger		Guilt		Fear		Shame		Disgust	
	Intensity	Duration	Intensity	Duration	Intensity	Duration	Intensity	Duration	Intensity	Duration	Intensity	Duration
Rumination	0.34***	0.25***	0.36***	0.39***	0.45***	0.39***	0.34***	0.38***	0.42***	0.33***	0.36***	0.47***
Reflection				0.10^†^	0.10^†^					0.26***		
Reappraisal												
Suppression												0.09^†^
Distraction				0.15**	0.10^†^	0.17**		0.16**				

*Note*. ***p<.001 **p<.01 *p<.05 ^†^ *p*<.1.

*Note*. Only significant regression weights are displayed in the table.

When predicting emotion intensity by all regulation strategies, it was found that rumination was positively associated with intensity for all emotions under study. However, the other four strategies did not consistently predict intensity. This may seem rather surprising as some of the other investigated strategies have been shown to be negatively related to intensity in previous research (e.g., reappraisal).

However, this lack of significant negative relationships may be explained by the existence of a reciprocal relation between regulation and emotion intensity [3], [9]: Regulation strategies do not only influence emotion intensity but intensity also influences regulation. Importantly, in contrast to the influence of regulation on intensity the influence of intensity on emotion regulation does not seem to depend much on the nature of the regulation strategy. Indeed, more intense experiences were found to initiate the use of a wide range of regulatory strategies [15]. As the influence from intensity to regulation is typically positive (more intense emotions require more regulation), one may find positive or null relationships between certain regulation strategies and intensity in correlational data, even when the influence of regulation on intensity is actually negative [9].

A possible way to deal with this is to control for the overall amount of emotion regulation [31]. Therefore, we ran a number of additional analyses in which intensity was predicted by a particular emotion regulation strategy and the mean use of all regulation strategies. Importantly, in these models, the regression weight of a particular emotion regulation strategy expresses the increase or decrease in intensity when this strategy is used relatively more (at the cost of the other strategies under study). Results from the additional analyses are presented in Table 6. To ease readability only significant regression weights are displayed in the table (the complete table can be found in Table S9 in File S2).

**Table 6:** Standardized Regression Weights for the Emotion-regulation strategies when Predicting Intensity and Duration of Emotion Episodes while Controlling for Mean Emotion Regulation.

	Sadness		Anger		Guilt		Fear		Shame		Disgust	
	Intensity	Duration	Intensity	Duration	Intensity	Duration	Intensity	Duration	Intensity	Duration	Intensity	Duration
Rumination	0.37***	0.24***	0.36***	0.37***	0.43***	0.34***	0.36***	0.31***	0.39***	0.36***	0.36***	0.47***
Reflection										0.28***	0.13^†^	0.15*
Reappraisal	−0.16*	−0.15*	−0.16*	−0.14*	−0.24***	−0.21**	−0.14*		−0.16*	−0.16*	−0.15*	
Suppression	−0.17**			−0.23***	−0.15*		−0.15*	−0.20**	−0.11^†^	−0.22***	−0.11^†^	−0.14*
Distraction	−0.12^†^						−0.11^†^			−0.20**	−0.14*	−0.27***

*Note*. ***p<.001 **p<.01 *p<.05 ^†^ *p*<.1.

*Note*. Only significant regression weights are displayed in the table.

From this table it can be seen that, for all emotions, rumination predicted intensity positively, and reappraisal predicted intensity negatively. This means that if rumination is used relatively more, intensity will increase, whereas if one reappraises relatively more, intensity will decrease. Furthermore it appeared that, for all emotions under study, except for anger, expressive suppression was negatively related to intensity. Also, distraction was negatively associated with the intensity of sadness, fear, and disgust. Finally, reflection did not predict intensity. In addition it appeared that the mean use of emotion-regulation strategies was typically significantly positively related to intensity, meaning that more intense emotion episodes were associated with more overall regulation.

When predicting emotion duration by all regulation strategies, it was found that, similar to intensity, for all emotions under study, rumination was positively related to duration. The other four strategies did not consistently predict duration. Again, this is most likely due to the reciprocal relation between duration and regulation. Therefore, similar to what was done for intensity, we controlled for the mean use of emotion regulation in subsequent analyses (see Table 6). It appears that, for all emotions, rumination positively predicted the duration of the episode, meaning that if one ruminates relatively more, the episode will last relatively long. Furthermore, suppression negatively predicted the duration of anger, fear, shame, and disgust episodes. Also, reappraisal negatively predicted the duration of sadness, anger, guilt, and shame episodes. Finally, distraction negatively predicted the duration of shame and disgust episodes, whereas reflection was positively related to the duration of these emotions. Again it appeared that, in general, the mean use of emotion-regulation strategies was positively related to duration, meaning that longer emotion episodes were associated with more overall regulation.

## Discussion

Intensity and duration are two central features of an emotional experience. Previous research revealed that emotion episodes display large variability in these features [4], [7]. In the present study, we systemically examined to what degree appraisals and emotion-regulation strategies can account for this variability. However, before addressing the results regarding this central research aim, we will briefly discuss our findings regarding (a) variability in intensity and duration between emotions, (b) variability in appraisals and regulation strategies between emotions, (c) the correlations between appraisals and regulation strategies and, (d) the correlation between emotion intensity and duration.

### Variability in Intensity and Duration between Emotions

Emotions differ in their average level of intensity and duration. Interestingly, a rather similar emotion order was found for intensity and duration. In particular, episodes of sadness were the most intense and lasted the longest, followed by anger, which in turn lasted longer and was more intense than episodes of guilt and fear. Episodes of shame and disgust lasted on average the shortest and were the least intense. This order is highly similar to the order that has been found in previous research on intensity [22] and duration [6], [17], [19]. One may conjecture that this order is explained by differences in appraisals between emotions: For example, the finding that episodes of sadness are elicited by events that are perceived as very important and hard to cope with may account for the relatively high intensity and long duration of sadness. Similarly, the finding that episodes of disgust are elicited by rather unimportant events that are easy to cope with may explain the rather low intensity and short duration of disgust.

### Variability in Appraisals and Regulation Strategies between Emotions

For both appraisals and regulation strategies evidence for variability between emotions was found. For appraisals, our findings match with previous research on appraisal theory: For example, anger and disgust have been associated with a violation of norms by someone else [32], [33]; accordingly, in the present study, episodes of these emotions were rated highest on other responsibility, injustice, and immorality. Also, sadness has been linked to low coping potential [34] and consistent with this, episodes of sadness were rated lowest on emotion-focused coping in the present study. Previously, fear has been linked with low emotion-focused coping [34], and in the present study, episodes of fear scored relatively low on this dimension compared to the other emotions under study. Finally, shame and guilt, two negative self-conscious emotions [35] were rated as having the most negative consequences for one’s self-image.

For regulation, it appeared that, for all strategies under study, regulatory effort was highest in case of sadness. This is interesting given that sadness was found to be a rather intense and long lasting emotion associated with low coping potential. This pattern of findings suggests that despite the low coping potential people try out a wide range of strategies but these strategies appear to be insufficient to compensate for the high levels of event importance that are characteristic for sadness. Furthermore, regulatory effort was the lowest for disgust, an emotion that was found to be rather low in intensity, short in duration and associated with high coping potential. This may reflect that participants are very efficient when regulating experiences of disgust, which are typically caused by rather unimportant events.

### The Relation between Appraisals and Emotion-regulation Strategies

Events that are appraised as important, disadvantageous, immoral, and having negatives consequences for one’s self image, were found to be positively associated with the use of a wide range of regulation strategies. As such, it seems that events that cause multiple mismatches (with one’s goals, norms or self-ideal) motivate the individual to take action to deal with the event and associated emotion. In contrast, emotion focused coping was found to be negatively related to the use of emotion regulation. High emotion focused coping may result from the feeling that no action is needed to deal with the emotion, which in turn results in a limited use of regulation strategies.

### The Relation between Emotion Intensity and Duration

Consistent with previous studies [5], [20], it appeared that emotion intensity and duration were only moderately correlated, with a median correlation of.38. This result is also consistent with a recent study [18] in which a median correlation between emotion intensity and duration of.39 was found. Interestingly, the lowest correlation was found for fear (*r* = .27). This difference between emotions may be explained by the manner in which emotions end [5], [18]: For example, fear episodes end rather often when the object of fear is removed (e.g., stage fright quickly dissipates when the performance is over) regardless of the intensity of the fear episode. For other negative emotions this seems to be less the case. These moderate correlations between intensity and duration imply that the relative predictive power of appraisals and emotion regulation may be different for emotion intensity and duration as discussed in the next section.

### Explaining Variability in Intensity and Duration: Appraisals and Regulation Strategies

Together, appraisals and regulation strategies explained about one third of the total variability in intensity and duration. A highly similar number was found for intensity in the study of Sonnemans and Frijda [9]. This result also implies that about two third of the total variability in intensity and duration was unexplained. A combination of several factors may be responsible for this result. First, in addition to appraisals and regulation strategies, other classes of predictors may play a role. In this context, it is notable that Frijda and colleagues argued that dispositions are another class of possible predictors of intensity [9]. However, it is not certain whether including this class would have substantially increased the percentage of explained variability given that in previous studies dispositions appeared to play a minor role compared to the two other classes of predictors [6], [9]. Second, although within each class of determinants, the most central predictors were included, some important variables may have been missing. For example, two frequently employed regulation strategies that were not investigated in the present study are social sharing [36] and self-distancing [37]. As such, including of a wider range of predictors within each class could also increase the percentage of variability explained.

Further, although both appraisals and regulation strategies were found to explain variability in intensity and duration, appraisals were found, as expected, to be stronger predictors of emotion intensity compared to emotion-regulation strategies. In contrast, for emotion duration, appraisals and regulation strategies were found to be more or less equally predictive. This confirms our expectation that regulation strategies are especially important to account for features reflecting emotional recovery such as emotion duration. However, it also shows that the role of appraisals is not restricted to the period of emotion elicitation and blossoming but that, instead, appraisals are predictive of emotion dynamics throughout the entire emotional episode. Interestingly, for both emotion duration and intensity, the predictive power of appraisals (regulation) strongly decreased when controlling for regulation (appraisals), suggesting that the effect of appraisals (regulation) on duration and intensity is partially mediated by regulation (appraisals).

Finally, we examined the relation between specific appraisals and regulation strategies on the one hand, and emotion intensity and duration on the other hand. Regarding appraisals, it was found that the importance of the emotion-eliciting event is positively related to both emotion intensity and duration. Furthermore, the study provided support for the mismatch hypothesis for both intensity and duration. For example, emotion-focused coping was negatively related with both the intensity and duration of negative emotions. However, the relation between appraisals and intensity differs to some extent from the relation between appraisals and duration. In this regard, it appeared that, across emotions, disadvantageousness of the emotion-eliciting event positively predicted intensity whereas duration was especially well predicted by the impact of the event on one’s self-image. These findings suggest that intensity is more strongly determined by the general adversity of the emotion-eliciting event, whereas duration depends more strongly on a specific form of adversity, that is, how negative the event is for one’s self-image. This may be explained by symbolic interactionist notions of the self [38] in which it is suggested that events that lead to a negative evaluation of the self are very pre-occupying (and so their impact may last longer) as they may require an adjustment of one’s self concept. Finally, it is notable that, in line with our predictions, expectedness and, own and other accountability were not related to intensity and duration of negative emotions.

Regarding regulation, a positive relation between rumination and emotion intensity and duration was found. Moreover, when controlling for the total amount of emotion regulation, an additional negative relationship between reappraisal and expressive suppression on the one hand and emotion intensity and duration on the other hand was found. Concerning rumination and reappraisal, these results are consistent with previous research showing these strategies to be maladaptive and adaptive, respectively [39]. For expressive suppression, the effect was not in line with our expectation that suppression would be a maladaptive strategy strengthening negative emotions. However, these results do line up with results from a recent meta-analysis [25] showing that expressive suppression may have positive consequences.

### Limitations of the Present Study

Although the present study provided some valuable insights regarding determinants of emotion intensity and duration, it is not without limitations. First, the collected data are cross-sectional in nature and, consequently, causal claims should be made carefully.

Second, participants were asked to report retrospectively on emotions. Even though the majority of reported episodes took place within the days and weeks preceding the study, part of the episodes took place longer ago. In previous studies, it has been shown that when participants are asked to report on emotional experiences that occurred recently, episodic information is used, whereas semantic knowledge is used when the emotional experience occurred a long time ago [40]. As a result, whereas estimates of the intensity and duration of recent emotions may reflect actual intensity and duration, estimates of the intensity and duration of emotions that occurred a long time ago may reflect perceived intensity and duration. However, even if semantic knowledge plays a role, this does not necessarily invalidate the intensity and duration estimates as according to the lexical sedimentation hypothesis [41] stable aspects of behavioral phenomena are encoded in lexical structure. Nevertheless, we performed a series of secondary analyses including only the episodes that occurred recently (days and weeks ago) and re-examined the relationship between appraisals and regulation strategies on the one hand, and emotion intensity and duration on the other hand. These analyses led to a pattern of results that was highly similar to the pattern obtained when all episodes were included. As such, the obtained conclusions are not strongly affected by the retrospective nature of the design.

Nevertheless, it may be worthwhile to replicate the current findings in future studies using online data collection methods such as experience sampling (ESM). An important advantage of these methods is that the collected data are not affected by any memory biases. However, it should be noted that it is not that straightforward to study emotion duration using an ESM approach. In particular, as the duration of an *uninterrupted* emotional episode is highly variable with durations ranging from a couple of seconds up until several hours, a typical ESM sampling rate may not be able to capture such a process very well (missing the exact onset, end, or even the total episode). Setting the sampling rate very high is also no perfect solution as this may give rise to artificial mental reappearances of the emotion-eliciting event, which have been shown to prolong the emotional experience [6].

Third, in the present study we focused on negative emotions. It would be interesting to extend the present findings to positive emotions, especially since in a number of recent studies researchers have started to investigate the appraisal basis of different positive emotions [42]. For positive emotions, a match hypothesis could be formulated, in that, events that are perceived as congruent with one’s goals, self-ideal, and values would elicit the most intense and long lasting positive emotions. Similarly, in some recent studies researchers have started to investigate the strategies that are used to regulate positive emotions [43] and it would be interesting to relate those strategies to both emotion intensity and duration.

## Supporting Information

File S1 **This file contains the Tables S1–S6 in which the correlations between appraisals and emotion-regulation strategies are displayed for each emotion separately.**
(DOCX)

File S2 **This file contains the Tables S7–S9.**
(DOCX)
