# Supplementary material for: Intensity and Duration of Negative Emotions: Comparing the Role of Appraisals and Regulation Strategies
Source: PLoS One. 2014 Mar 26;9(3):e92410. doi: 10.1371/journal.pone.0092410 (PMC3966809; doi:10.1371/journal.pone.0092410)
Supplement: File S1 — This file contains the Tables S1–S6 in which the correlations between appraisals and emotion-regulation strategies are displayed for each emotion separately. (DOCX) [file pone.0092410.s001.docx]

Table S1. Correlations between Appraisals and Emotion-regulation Strategies for Episodes of Anger.

|  | Rumination | Reflection | Reappraisal | Suppression | Distraction |
| --- | --- | --- | --- | --- | --- |
| Importance | .50^**^ | .35^**^ | .20^**^ | .18^**^ | .26^**^ |
| Disadvantageousness | .31^**^ | .27^**^ | .19^**^ | .24^**^ | .18^**^ |
| Other responsibility | -.01 | .05 | .04 | -.06 | -.03 |
| Own responsibility | .16^**^ | .14^**^ | .21^**^ | .15^**^ | .12^*^ |
| Problem-focused coping | .00 | .00 | .08 | .04 | .05 |
| Emotion-focused coping | -.36^**^ | -.22^**^ | -.12^*^ | -.10^*^ | -.21^**^ |
| Expectedness | -.05 | -.02 | .06 | -.02 | .03 |
| Injustice | .26^**^ | .17^**^ | .13^*^ | .03 | .06 |
| Self-image | .53^**^ | .41^**^ | .35^**^ | .32^**^ | .35^**^ |
| Immorality | .29^**^ | .20^**^ | .17^**^ | .17^**^ | .19^**^ |
| ** *p* < 0.01 * *p* < 0.05 | | | | | |

**Table S2. Correlations between Appraisals and Emotion-regulation Strategies for Episodes of Disgust.**

|  | Rumination | Reflection | Reappraisal | Suppression | Distraction |
| --- | --- | --- | --- | --- | --- |
| Importance | .65^**^ | .53^**^ | .35^**^ | .27^**^ | .17^**^ |
| Disadvantageousness | .39^**^ | .28^**^ | .19^**^ | .22^**^ | .28^**^ |
| Other responsibility | .08 | .13^**^ | .01 | .01 | .17^**^ |
| Own responsibility | .11^*^ | .06 | .15^**^ | .11^*^ | -.01 |
| Problem-focused coping | .10^*^ | .13^*^ | .05 | .06 | -.03 |
| Emotion-focused coping | -.44^**^ | -.30^**^ | -.27^**^ | -.21^**^ | -.20^**^ |
| Expectedness | -.10 | .03 | .04 | .07 | .00 |
| Injustice | .50^**^ | .42^**^ | .23^**^ | .08 | .09 |
| Self-image | .62^**^ | .52^**^ | .40^**^ | .36^**^ | .25^**^ |
| Immorality | .45^**^ | .38^**^ | .21^**^ | .10^*^ | .08 |
| ** *p* < 0.01 * *p* < 0.05 | | | | | |

**Table S3. Correlations between Appraisals and Emotion-regulation Strategies for Episodes of Fear.**

|  | Rumination | Reflection | Reappraisal | Suppression | Distraction |
| --- | --- | --- | --- | --- | --- |
| Importance | .54^**^ | .38^**^ | .22^**^ | .10^*^ | .16^**^ |
| Disadvantageousness | .38^**^ | .26^**^ | .11^*^ | .14^**^ | .15^**^ |
| Other responsibility | .12^*^ | .12^*^ | -.04 | -.06 | -.02 |
| Own responsibility | .13^*^ | .15^**^ | .19^**^ | .21^**^ | .09 |
| Problem-focused coping | .08 | .09 | .11^*^ | .14^**^ | .06 |
| Emotion-focused coping | -.28^**^ | -.13^**^ | -.11^*^ | -.10 | -.16^**^ |
| Expectedness | -.08 | -.03 | .09 | .22^**^ | .19^**^ |
| Injustice | .36^**^ | .30^**^ | .17^**^ | .10 | .18^**^ |
| Self-image | .44^**^ | .37^**^ | .28^**^ | .22^**^ | .17^**^ |
| Immorality | .33^**^ | .31^**^ | .17^**^ | .14^**^ | .18^**^ |
| ** *p* < 0.01 * *p* < 0.05 | | | | | |

**Table S4. Correlations between Appraisals and Emotion-regulation Strategies for Episodes of Guilt.**

|  | Rumination | Reflection | Reappraisal | Suppression | Distraction |
| --- | --- | --- | --- | --- | --- |
| Importance | .47^**^ | .33^**^ | .22^**^ | .18^**^ | .28^**^ |
| Disadvantageousness | .32^**^ | .19^**^ | .16^**^ | .15^**^ | .23^**^ |
| Other responsibility | .15^**^ | .21^**^ | .21^**^ | .15^**^ | .10^*^ |
| Own responsibility | .09 | -.03 | -.05 | .05 | .04 |
| Problem-focused coping | .17^**^ | .17^**^ | .12^*^ | .13^**^ | .12^*^ |
| Emotion-focused coping | -.29^**^ | -.10 | -.13^**^ | -.20^**^ | -.28^**^ |
| Expectedness | .06 | .06 | .13^*^ | .12^*^ | .06 |
| Injustice | .27^**^ | .12^*^ | .17^**^ | .18^**^ | .16^**^ |
| Self-image | .51^**^ | .39^**^ | .37^**^ | .34^**^ | .45^**^ |
| Immorality | .32^**^ | .25^**^ | .21^**^ | .26^**^ | .28^**^ |
| ** *p* < 0.01 * *p* < 0.05 | | | | | |

**Table S5. Correlations between Appraisals and Emotion-regulation Strategies for Episodes of Sadness.**

|  | Rumination | Reflection | Reappraisal | Suppression | Distraction |
| --- | --- | --- | --- | --- | --- |
| Importance | .30^**^ | .16^**^ | .07 | .08 | .09 |
| Disadvantageousness | .34^**^ | .20^**^ | .06 | .12^*^ | .18^**^ |
| Other responsibility | .07 | .11^*^ | .05 | .06 | .00 |
| Own responsibility | .12^*^ | .11^*^ | .19^**^ | .09 | .04 |
| Problem-focused coping | .14^**^ | .13^**^ | .15^**^ | .09 | .02 |
| Emotion-focused coping | -.38^**^ | -.12^*^ | .05 | -.04 | -.11^*^ |
| Expectedness | -.07 | -.01 | .00 | .06 | .14^**^ |
| Injustice | .26^**^ | .08 | -.01 | .11^*^ | .06 |
| Self-image | .32^**^ | .17^**^ | .21^**^ | .19^**^ | .09 |
| Immorality | .15^**^ | .11^*^ | .07 | .05 | .01 |
| ** *p* < 0.01 * *p* < 0.05 | | | | | |

**Table S6. Correlations between Appraisals and Emotion-regulation Strategies for Episodes of Shame.**

|  | Rumination | Reflection | Reappraisal | Suppression | Distraction |
| --- | --- | --- | --- | --- | --- |
| Importance | .53^**^ | .48^**^ | .36^**^ | .25^**^ | .28^**^ |
| Disadvantageousness | .48^**^ | .33^**^ | .26^**^ | .31^**^ | .32^**^ |
| Other responsibility | .12^*^ | .16^**^ | .16^**^ | .08 | .15^**^ |
| Own responsibility | .12^*^ | .11^*^ | -.01 | .11^*^ | .05 |
| Problem-focused coping | .17^**^ | .17^**^ | .09 | .17^**^ | .14^**^ |
| Emotion-focused coping | -.38^**^ | -.29^**^ | -.13^*^ | -.16^**^ | -.11^*^ |
| Expectedness | .12^*^ | .20^**^ | .16^**^ | .14^**^ | .10^*^ |
| Injustice | .25^**^ | .20^**^ | .17^**^ | .23^**^ | .17^**^ |
| Self-image | .56^**^ | .37^**^ | .30^**^ | .37^**^ | .36^**^ |
| Immorality | .30^**^ | .31^**^ | .24^**^ | .22^**^ | .19^**^ |
| ** *p* < 0.01 * *p* < 0.05 | | | | | |
