# Supplementary material for: Intensity and Duration of Negative Emotions: Comparing the Role of Appraisals and Regulation Strategies
Source: PLoS One. 2014 Mar 26;9(3):e92410. doi: 10.1371/journal.pone.0092410 (PMC3966809; doi:10.1371/journal.pone.0092410)
Supplement: File S2 — This file contains the Tables S7–S9. (DOCX) [file pone.0092410.s002.docx]

Table S7. Standardized Regression Weights for the Appraisals when Predicting the Intensity and Duration of Emotion Episodes.

|  |  | Sadness | | Anger | | Guilt | | Fear | | Shame | | Disgust | |
| --- | --- | --- | --- | --- | --- | --- | --- | --- | --- | --- | --- | --- | --- |
|  |  | Intensity | Duration | Intensity | Duration | Intensity | Duration | Intensity | Duration | Intensity | Duration | Intensity | Duration |
| Importance |  | 0.35^***^ | 0.21^***^ | 0.36^***^ | 0.28^***^ | 0.37^***^ | 0.29^***^ | 0.31^***^ | 0.31^***^ | 0.22^***^ | 0.32^***^ | 0.39^***^ | 0.38^***^ |
| Disadvantage |  | 0.13^*^ | 0.26^***^ | 0.12^*^ | 0.08 | 0.10^*^ | 0.06 | 0.20^***^ | 0.08 | 0.15^**^ | 0.02 | 0.03 | 0.05 |
| Responsibility |  |  |  |  |  |  |  |  |  |  |  |  |  |
| Other |  | -0.06 | -0.12^*^ | 0.02 | -0.02 | 0.01 | 0.03 | 0.06 | -0.05 | 0.08 | 0.06 | 0.06 | -0.01 |
| Own |  | 0.03 | -0.07 | 0.05 | 0.00 | 0.03 | -0.04 | 0.01 | 0.03 | -0.02 | 0.04 | 0.04 | 0.03 |
| Coping |  |  |  |  |  |  |  |  |  |  |  |  |  |
| Problem-foc. |  | -0.08 | -0.15^**^ | 0.03 | -0.06 | 0.12^**^ | 0.04 | -0.03 | -0.10^†^ | 0.07 | 0.00 | -0.09^†^ | -0.06 |
| Emotion- foc. |  | -0.17^***^ | -0.15^**^ | -0.25^***^ | -0.12^*^ | -0.14^**^ | -0.19^***^ | -0.17^***^ | -0.04 | -0.08 | -0.15^**^ | -0.03 | 0.09^†^ |
| Expectedness |  | 0.01 | 0.07 | -0.03 | 0.04 | -0.03 | -0.05 | -0.02 | 0.11^*^ | -0.08^†^ | 0.03 | -0.03 | -0.05 |
| Injustice |  | 0.11^*^ | 0.12^*^ | 0.07 | 0.03 | -0.01 | 0.01 | 0.02 | 0.05 | 0.03 | 0.04 | -0.01 | 0.05 |
| Self image |  | 0.05 | 0.03 | -0.04 | 0.13^*^ | 0.16^**^ | 0.10^†^ | -0.04 | 0.12^*^ | 0.18^**^ | 0.12^*^ | -0.02 | 0.20^**^ |
| Immorality |  | -0.02 | 0.09^†^ | 0.04 | 0.09^†^ | 0.12^*^ | 0.07 | -0.01 | 0.07 | 0.05 | 0.11^*^ | 0.10 | 0.08 |
| *Note*. ^***^*p* < .001 ^**^*p* < .01 ^*^*p* < .05 ^†^ *p* < .1 | | | | | | | | | | | | | |

Table S8. Standardized Regression Weights for the Emotion Regulation Strategies when Predicting Intensity and Duration of Emotion Episodes.

|  | Sadness | | Anger | | Guilt | | Fear | | Shame | | Disgust | |
| --- | --- | --- | --- | --- | --- | --- | --- | --- | --- | --- | --- | --- |
|  | Intensity | Duration | Intensity | Duration | Intensity | Duration | Intensity | Duration | Intensity | Duration | Intensity | Duration |
| Rumination | 0.34^***^ | 0.25^***^ | 0.36^***^ | 0.39^***^ | 0.45^***^ | 0.39^***^ | 0.34^***^ | 0.38^***^ | 0.42^***^ | 0.33^***^ | 0.36^***^ | 0.47^***^ |
| Reflection | 0.06 | 0.04 | 0.06 | 0.10^†^ | 0.10^†^ | 0.05 | 0.05 | 0.00 | 0.02 | 0.26^***^ | 0.08 | 0.05 |
| Reappraisal | -0.05 | -0.03 | -0.07 | -0.03 | -0.05 | -0.03 | -0.08 | 0.09 | 0.00 | -0.04 | -0.07 | 0.01 |
| Suppression | -0.06 | 0.03 | 0.02 | -0.08 | 0.03 | 0.04 | -0.02 | -0.05 | 0.03 | 0.00 | 0.06 | 0.09^†^ |
| Distraction | 0.01 | 0.05 | 0.02 | 0.15^**^ | 0.10^†^ | 0.17^**^ | 0.03 | 0.16^**^ | 0.06 | . 003 | 0.02 | -0.06 |
| *Note*. ^***^*p* < .001 ^**^*p* < .01 ^*^*p* < .05 ^†^*p* < .1 | | | | | | | | | | | | |

Table S9. Standardized Regression Weights for the Emotion Regulation Strategies when Predicting Intensity and Duration of Emotion Episodes while Controlling for Mean Emotion Regulation.

|  | Sadness | | Anger | | Guilt | | Fear | | Shame | | Disgust | |
| --- | --- | --- | --- | --- | --- | --- | --- | --- | --- | --- | --- | --- |
|  | Intensity | Duration | Intensity | Duration | Intensity | Duration | Intensity | Duration | Intensity | Duration | Intensity | Duration |
| Rumination | 0.37^***^ | 0.24^***^ | 0.36^***^ | 0.37^***^ | 0.43^***^ | 0.34^***^ | 0.36^***^ | 0.31^***^ | 0.39^***^ | 0.36^***^ | 0.36^***^ | 0.47^***^ |
| Reflection | 0.09 | .01 | 0.05 | 0.08 | 0.07 | -0.03 | 0.08 | -0.02 | 0.01 | 0.28^***^ | 0.13^†^ | 0.15^*^ |
| Reappraisal | -0.16^*^ | -0.15^*^ | -0.16^*^ | -0.14^*^ | -0.24^***^ | -0.21^**^ | -0.14^*^ | -0.04 | -0.16^*^ | -0.16^*^ | -0.15^*^ | -0.07 |
| Suppression | -0.17^**^ | -0.06 | -0.10 | -0.23^***^ | -0.15^*^ | -0.09 | -0.15^*^ | -0.20^**^ | -0.11^†^ | -0.22^***^ | -0.11^†^ | -0.14^*^ |
| Distraction | -0.12^†^ | -0.04 | -0.10 | -0.01 | -0.09 | 0.03 | -0.11^†^ | -0.02 | -0.09 | -0.20^**^ | -0.14^*^ | -0.27^***^ |
| *Note*. ^***^*p* < .001 ^**^*p* < .01 ^*^*p* < .05 ^†^*p* < .1 | | | | | | | | | | | | |
